# Effect of Distributed Mass on the Node, Frequency, and Sensitivity of Resonant-Mode Based Cantilevers

**DOI:** 10.3390/s17071621

**Published:** 2017-07-13

**Authors:** Kewei Zhang, Qianke Zhu, Zhe Chen

**Affiliations:** School of Materials Science and Engineering, Taiyuan University of Science and Technology, Taiyuan 030024, China; zhuqianke@gmail.com (Q.Z.); chenzhe010@gmail.com (Z.C.)

**Keywords:** cantilever, mass load, node, frequency shift, sensitivity

## Abstract

We derived an analytical expression for a resonant-mode based bi-layered cantilever with distributed mass load. The behavior of mode of vibration, nodal position, frequency shift, as well as sensitivity under different mass load distributions was theoretically studied. The theoretical results suggested that asymmetric mass load distribution leads to the shift of nodes as well as the sensitive regions of a resonant-mode based cantilever. *n* − 1 local maximal sensitivities and *n* − 1 local minimal sensitivities are observed when the cantilever vibrates in the nth-order resonance. The maximal sensitivity is found at the first local maximal sensitivity and the behavior of mass load length as a function of the maximal sensitivity follows the rule of an exponent decaying function. The sensitivity increases as the load mass increases for the same mass load distribution, but the corresponding slopes are different.

## 1. Introduction

In the past decades, resonant-mode based cantilevers have shown high potential for the bio/chemical sensing applications [1,2,3,4]. The working principle is based on the shift in the resonant frequency due to the mass load caused by the specific binding of targets (i.e., microorganism, chemicals) on their surfaces [5,6,7]. For any type of sensors, sensitivity is an important parameter to characterize their performance. The sensitivity (*S_m_*) for a resonant-mode based cantilever sensor is defined as its change in resonant frequency due to each unit of mass load, that is, *S_m_* = −Δ*f*/Δ*m* [8]. A resonant-mode based cantilever can be single-layered or multi-layered in structure depending on the beam materials and actuation mechanism [8]. Mathematically, a multi-layered cantilever can be treated as a single-layered cantilever with equivalent mechanical properties. The equations for the sensitivity of a single-layered cantilever in a flexural vibration mode in the following two cases have been known as

Case I: Targets are uniformly bound on the entire surface of a cantilever [9].

(1) $$S_{m,uni}=\frac{1}{4}\frac{\lambda_{n}^{2}}{\pi \sqrt{12}}\frac{h}{WL^{2}}\sqrt{\frac{E}{\rho^{3}(1-\sigma^{2})}}$$

Case II: Targets are bound at the free end of a cantilever [10].

(2) $$S_{m,tip}=\frac{1}{0.944}\frac{\lambda_{n}^{2}}{\pi \sqrt{12}}\frac{1}{WL^{2}}\sqrt{\frac{E}{\rho^{3}(1-\sigma^{2})}}$$

where *λ_n_* is the dimensionless *n*th-mode Eigenvalue; *h*, *W*, *L* are the thickness, width, and length of the cantilever; *E*, *ρ*, and *σ* are the effective Young’s modulus, density, and the Poisson’s ratio of the beam material; Δ*m* and *M* are the mass of the load and the cantilever itself (Δ*m*<<*M*).

It is found that sensitivity is inversely proportional to the cantilever size. However, the signal would be too weak to be detected when the size is very small due to the limitation of measurement devices. From the viewpoint of material properties, a resonant-mode based cantilever with higher Young’s modulus and lower density can also increase the sensitivity. However, other material properties such as coupling coefficient and *Q* value also need to be considered, which would make the selection of the cantilever material difficult. Another way to increase the sensitivity is to use higher-order resonance modes [11,12,13,14]. However, there is also a concern about the signal strength. For the same cantilever, the sensitivity in case II is much higher than that in case I which means that target distribution has a strong effect on the sensitivity of a resonant-mode based cantilever. Therefore, besides decreasing the size and replacing materials, controlling the target (i.e., mass load) distribution is a practical way to increase the sensitivity of a resonant-mode based cantilever.

Some work has been done on studying the effect of mass load conditions on resonant frequency and sensitivity of resonant-mode based cantilevers. Yi et al. investigated, both experimentally and theoretically, the resonance frequency of a piezoelectric single-layered cantilever with point mass loaded at the tip of the cantilever [15]. Dohn et al., derived an analytical expression for the resonant frequency of a cantilever with point mass load in different positions [16]. Maraldo et al., experimentally investigated the variation of sensitivity of a piezoelectric bi-layered cantilever with the change of point mass position and magnitude [17]. In the previous study, we established a theoretical equation for a bi-layered cantilever attached with point mass and investigated the effect of loading position and mass magnitude on the sensitivity of the cantilever using a modal analysis method [18]. In this work, a further theoretical investigation on the mode of vibration, node, resonant frequency, and sensitivity of a bi-layered resonant-mode based cantilever with distributed mass load was done aiming at development of a highly sensitive cantilever by controlling the target distribution.

## 2. Theory and Derivation

**Figure 1:**
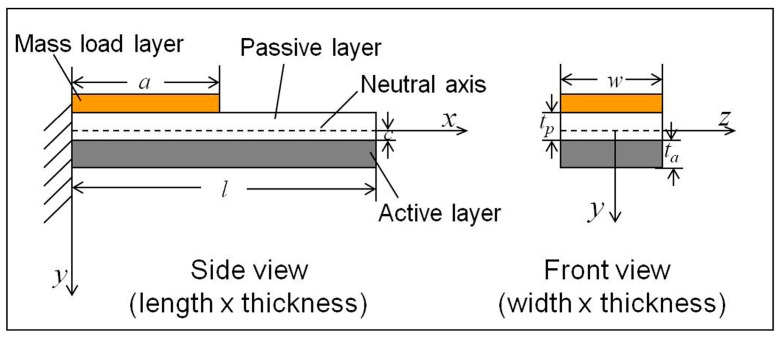
Schematic illustration of a resonant-mode based cantilever with a layer of distributed mass load on its surface. *l* and *w* are the length and width of the cantilever beam; *t*_p_ and *t*_a_ are the thickness of the passive layer and active layer; *a* is length of the mass layer; *x*-axis is established on the neutral axis; *c* is the distance between *x*-axis and the interface of the two layers.

A bi-layered cantilever is composed of an active layer made of smart materials (i.e., magnetostrictive materials, piezoelectric materials) and a passive layer made of structural materials. Due to magnetostriction or piezoelectric effect of the active layer and restriction of the structure layer, the cantilever vibrates in a flexural mode when subjected to an alternative magnetic or electric field along the beam length direction. A bi-layered cantilever with a layer of distributed mass load is shown in Figure 1.

The kinetic energy (*T*) and potential energy (*V*) of the bi-layered cantilever as shown in Figure 1 are expressed as

(3) $$T=\frac{1}{2}\int_{0}^{l}\rho_{0}A_{0}{\left(\frac{\partial u\left(x,t\right)}{\partial t}\right)}^{2}dx+\frac{1}{2}\int_{0}^{a}\rho_{m}A_{m}{\left(\frac{\partial u\left(x,t\right)}{\partial t}\right)}^{2}dx$$

(4) $$V=\frac{1}{2}\int_{0}^{l}E_{0}I_{0}\left(\frac{\partial^{2}u\left(x,t\right)}{\partial x^{2}}\right)dx$$

where *A*_0_ = *A_a_* + *A_p_*; $\rho_{0}=\frac{\rho_{a}A_{a}+\rho_{p}A_{p}}{A_{a}+A_{p}}$; $E_{0}I_{0}=\frac{E_{a}}{1-\nu_{a}^{2}}I_{a}+\frac{E_{p}}{1-\nu_{p}^{2}}I_{p}$; *A_a_*, *ρ_a_*, *E_a_*, *v_a_*, and *I_a_* are the cross-sectional area, density, Young’s modulus, Poisson’s ratio, and moment of inertia of the active layer; *A_p_*, *ρ_p_*, *E_p_*, *v_p_*, and *I_p_* are the cross-sectional area, density, Young’s modulus, Poisson’s ratio, and moment of inertia of the passive layer; $u(x,t)=\sum_{i=1}^{n}\varphi_{i}(x)q_{i}(t)$ where *φ_i_*(*x*) is the mode shape function and *q_i_*(*t*) is the generalized coordinate.

Equations (3) and (4) can be further simplified as

(5) $$T=\frac{1}{2}{\dot{q}}^{T}(M_{0}+M_{1})\dot{q}$$

(6) $$V=\frac{1}{2}q^{T}Kq$$

where $M_{0}=\int_{0}^{l}\rho_{0}A_{0}\varphi_{i}^{}\left(x\right)\varphi_{j}^{}\left(x\right)dx$; *M*_1_ = *ρ_m_A_m_φ_i_*(*x*)*φ_j_*(*x*)d*x*; *M* = *M*_0_ + *M*_1_; $K=\int_{0}^{l}E_{0}I_{0}\left(\frac{\partial^{2}\varphi_{i}}{\partial x^{2}}\right)\left(\frac{\partial^{2}\varphi_{j}}{\partial x^{2}}\right)dx$.

It is known that the mode shape function $\varphi_{n}=\sin\beta_{n}x-\sinh\beta_{n}x-\alpha_{n}(\cos\beta_{n}x-\cosh\beta_{n}x)$ [9] (where $\alpha_{n}=\frac{\sin\beta_{n}l+\sinh\beta_{n}l}{\cosh\beta_{n}x+\cos\beta_{n}x}$; *n* = 1, 2, 3,… and the values of *β_n_* for the first four order modes are *β*_1_ = 1.87510/*l*, *β*_2_ = 4.69410/*l*, *β*_3_ = 7.85476/*l*, *β*_4_ = 10.99554/*l*, respectively) satisfies the boundary conditions of a single-layered cantilever

Combining Equations (4) and (5) and Lagrange’s Equation, the governing vibration equation of the cantilever with distributed mass load is given by

(7) $$(K-\omega_{n}^{2}M)q=0$$

Obviously, the resonant frequency and mode of vibration of a cantilever is dependent on *K* and *M* which are affected by mass load distribution. By solving Equation (7), *q* and *ω_n_* are determined and the resonant frequency *f_n_* is calculated by

*f_n_* = *ω_n_*/2*π* (8)

Finally, sensitivity *S_m_*_,*n*_ is obtained by

(9) $$S_{m,n}=\frac{f_{n,0}-f_{n,m}}{\Delta m}$$

where Δ*m* is the load mass; *f_n_*_,0_ and *f_n_*_,*m*_ are the *n*th-order resonant frequency of the cantilever without and with mass load.

## 3. Materials and Mass Load Conditions

In this study, the commercially available magnetostrictive alloy Metglas^TM^ 2826 (Metglas Inc., Conway, SC, USA) is selected as the active layer and copper is selected as the passive layer. The material properties and mass load conditions are listed in Table 1.

## 4. Results and Discussion

### 4.1. Effecton Modes of Vibration and Nodal Positions

**Figure 2:**
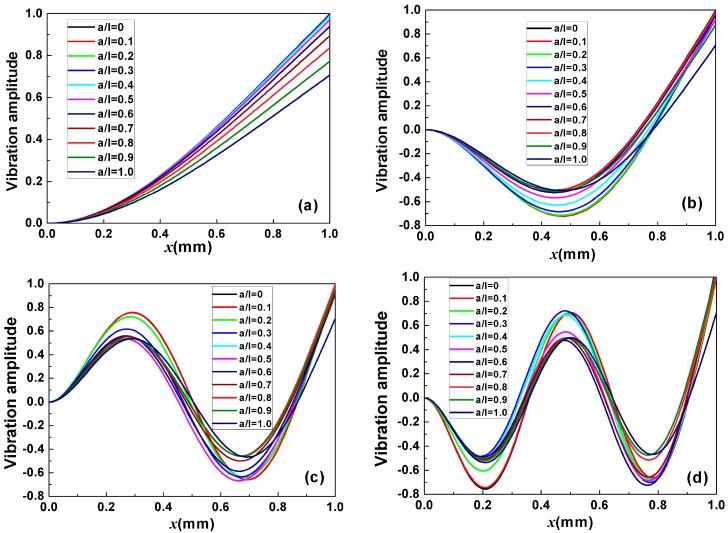
The mode of vibration of the cantilever with different mass load distribution (*a*/*l*) under: (**a**) First-order resonance mode; (**b**) Secnd-order resonance mode; (**c**) Third-order resonance mode; (**d**) Fourth-order resonance mode where *ρ_m_A_m_*/*ρ*_0_*A*_0_ = 1.

Figure 2 shows the behavior of mode of vibration of the cantilever with different mass load distribution (*a*/*l*). Disregarding the fixed end, *n* − 1 nodes are observed for the *n*th-order resonance mode. Moreover, the change in mode of vibration with mass load distribution causes the nodes to shift (i.e., the difference between the nodal position *x* of the cantilever with mass load *a*/*l* ≠ 0 and without mass load *a*/*l* = 0) to the fixed end of the cantilever beam.

**Figure 3:**
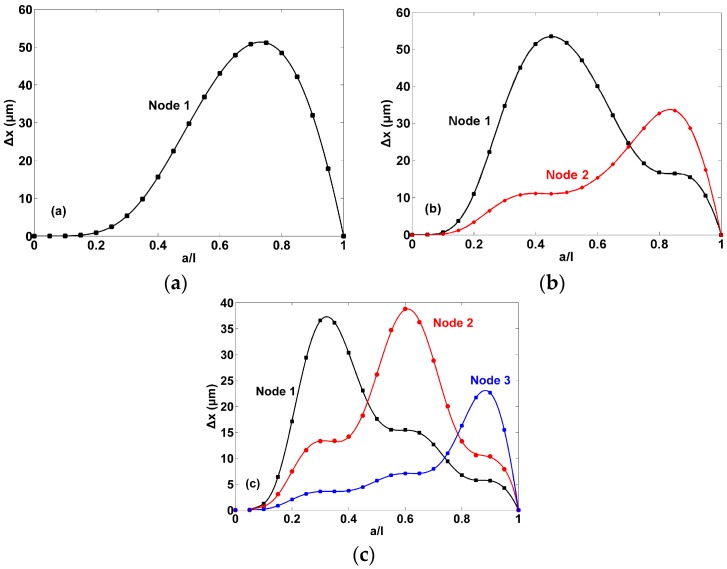
Behavior of nodal shift Δ*x* of the cantilever with different mass load distribution (*a*/*l*) under: (**a**) Second-order resonance; (**b**) Third-order resonance; (**c**) Fourth-order resonance where *ρ_m_A_m_*/*ρ*_0_*A*_0_ = 1.

To further investigate the behavior of nodal position under different mass load distributions, the nodal shift Δ*x* as a function of *a*/*l* is plotted as shown in Figure 3. Clearly, for all the nodes, the nodal shift firstly increases to the maximum and then decreases to zero as *a*/*l* increases to 1. Since *a*/*l* = 1 means that the mass is uniformly and symmetrically distributed on the entire surface of the cantilever, we can conclude that asymmetric mass load distributions (i.e., *a*/*l* ≠ 1) cause the nodes to shift. However, the value of nodal shift is not related to the degree of symmetry of mass distribution. For example, for the second-order resonance, the sequence of *a*/*l* causing the nodes shift from the largest to the smallest is *a*/*l* = 0.7, 0.8, 0.6, 0.9, 0.5, 0.4, 0.3, 0.2, 0.1. Moreover, the value of *a*/*l* corresponding to the maximal nodal shift is found near the nodal position and decreases with the resonance order increasing for the same node. For example, the value of *a*/*l* corresponding to the maximal shift for node 1 in the second-order, third-order, and fourth-order resonances are ~0.75, ~0.45, and ~0.3, respectively.

### 4.2. Effecton Resonant Frequency

**Figure 4:**
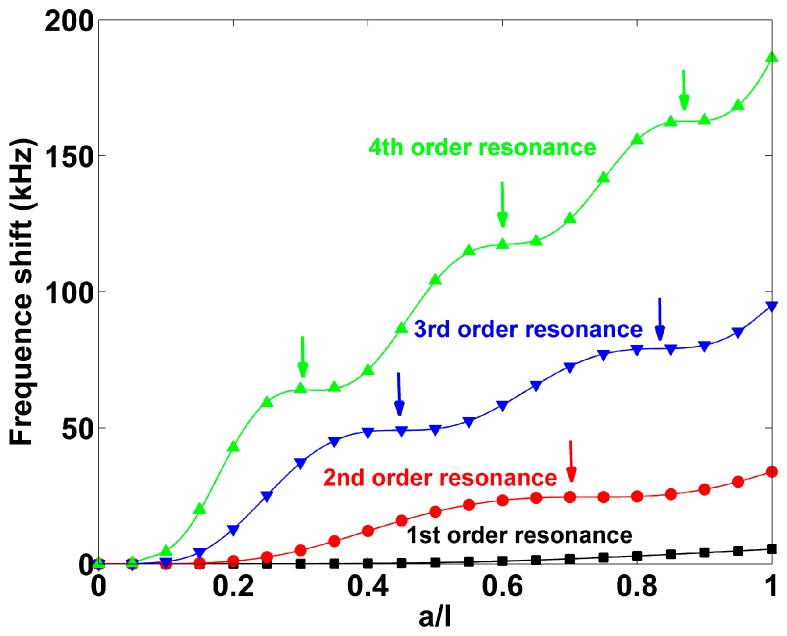
The behavior of frequency shift of the cantilever with different mass distribution where *ρ_m_A_m_*/*ρ*_0_*A*_0_ = 1.

Figure 4 shows frequency shift of the cantilever as a function of mass load distribution under different order resonances. It can be seen that the frequency shift increases as *a*/*l* increases for each resonance mode. However, the curves are nearly flat when the values of *a*/*l* fall in some specific ranges as marked by the colored arrows. In the other words, mass loaded in these regions has little contribution on frequency shift. It is worth noting that the nodal number is the same as that of the flat regions and each nodal positionnearly corresponds to the middle of a flat region. This phenomenon is very similar with the experimental results for the cantilevers reported by Johnson, B.N. et al. [22] and that of free-standing mangetostricitive sensors in longitudinal vibration mode [23]. Particularly, when the mass is loaded at a node, there would be no effect on resonant frequency shift. Therefore, it is believed that the closer the mass is loaded to the node of the cantilever, the less is contributed to the frequency shift and vice versa. Since the nodal positions shift as mass load distribution changes, the mass sensitive regions shift as the nodes move.

### 4.3. Effecton Sensitivity

**Figure 5:**
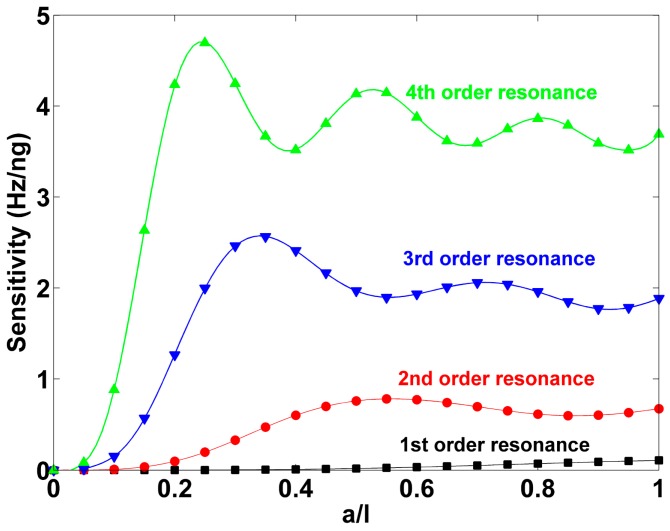
The behavior of sensitivity of the cantilever as the change of mass distribution under different resonance modes where *ρ_m_A_m_*/*ρ*_0_*A*_0_ = 1.

Figure 5 shows sensitivity (*S_m_*) of the cantilever as a function of mass distribution under different resonance modes. For the first order resonance, the sensitivity increases slowly at beginning and then increases dramatically as *a*/*l* increases. For the higher order resonance modes, the sensitivity first increases to the maximum and then fluctuates with decaying amplitude as *a*/*l* increases which results in *n* − 1 peaks (local maximum) and *n* − 1 valleys (local minimum) for the cantilever vibrating in the *n*th-order resonance and these values are found to be the points of inflection of the curves in Figure 4. Below the value of *a*/*l* for the maximal sensitivity, the higher the resonance order, the faster the sensitivity increases as *a*/*l*. It is also found that the value of *a*/*l* for each local minimum is close to, but slightly larger than, the corresponding nodal position. Taking the fourth-order resonance as an example, the values of *a*/*l* for the three minimal sensitivities are ~0.4, ~0.7, and ~0.95 while the three nodal positions are ~0.36, ~0.64, and ~0.91, respectively.

**Figure 6:**
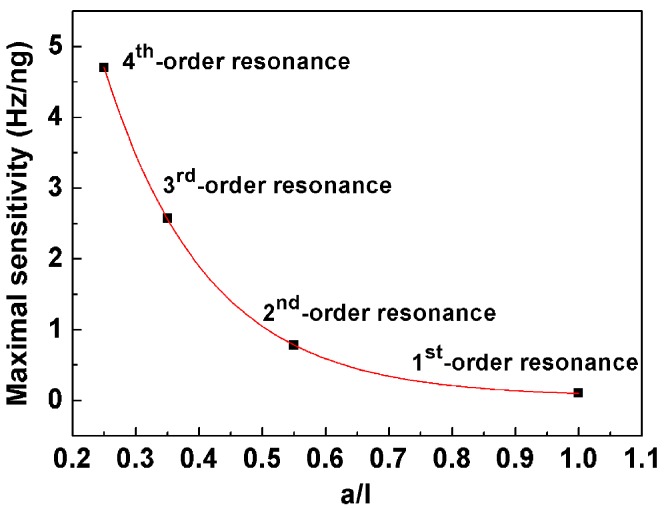
The maximal sensitivity as a function of mass load distribution (*a*/*l*).

Moreover, *a*/*l* for the maximal sensitivity shifts to the fixed end as the resonance order increases as shown in Figure 6. It is found that the data curve can be fitted well by an exponent decaying function as follows

*S*_*m*,max_ = *y*_0_ + *Ae*^(−*x*/*c*)^ (10)

where *y*_0_ = 0.05842, *A* = 21.72383, *c* = 1.62126 × 10^−4^; *x* represents *a*/*l*.

Therefore, *a*/*l* corresponding to the maximal sensitivity of the cantilever under higher order resonances can be predicted, based on Equation (10).

**Figure 7:**
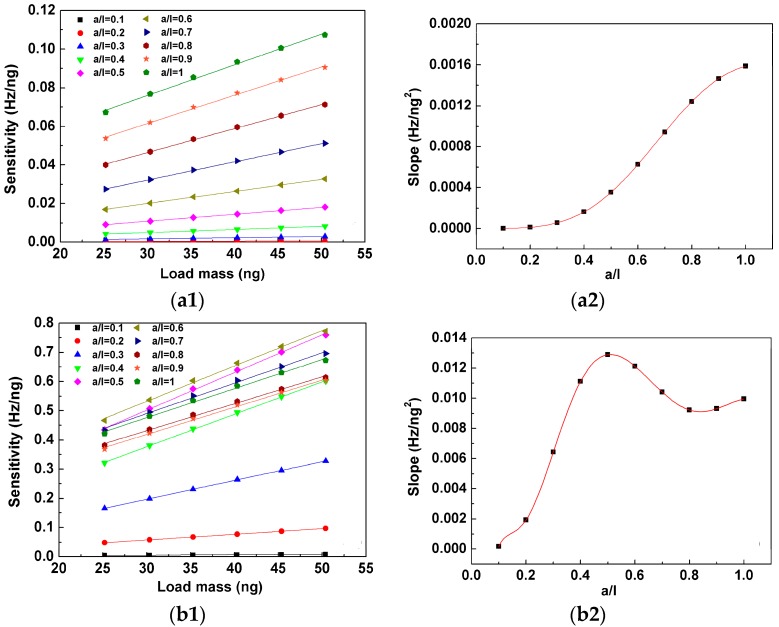

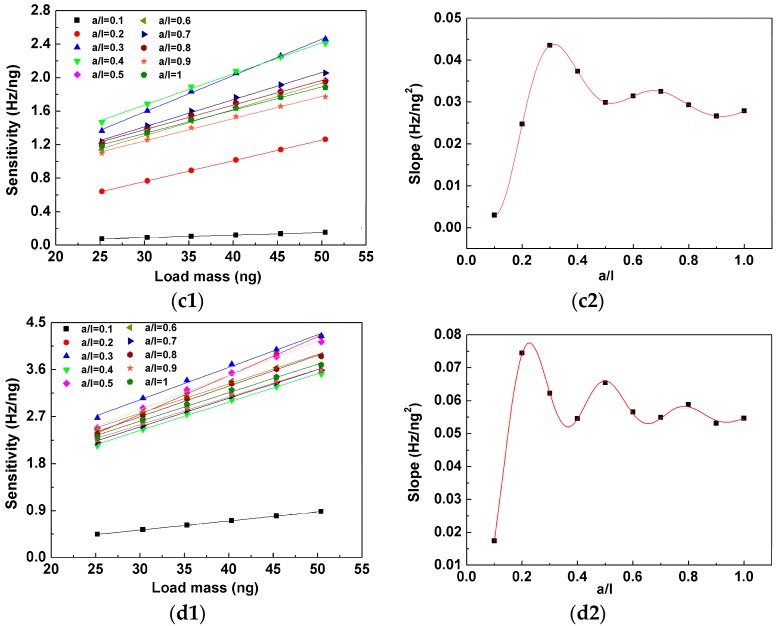
Sensitivity of the cantilever as a function of load mass under (**a1**) first-order resonance, (**b1**) second-order resonance, (**c1**) third-order resonance, and (**d1**) fourth-order resonance. All the data points are fitted by linear functions and the slopes of the linear fittings as a function of *a*/*l* are shown in Figure 7(**a2**,**b2**,**c2**,**d2**).

The behavior of sensitivity of the cantilever with mass load in six different density ratios (i.e., *ρ_m_A_m_*/*ρ*_0_*A*_0_ = 0.5, 0.6, 0.7, 0.8, 0.9, 1) for the first four order resonances are shown in Figure 7(a1,d1). It can be seen that the sensitivity is linearly proportional to load mass for the same mass distribution *a*/*l* under the same order resonance but the slopes are different as the value of *a*/*l* changes as shown in Figure 7(a2,d2). It is worth noting that the behavior of the slopes as a function of *a*/*l* is very similar with that of the sensitivity in the same order resonance as shown in Figure 5 but the corresponding peaks and valleys are not in the same positions except for the first order resonance. In other words, the maximal sensitivity does not increase the fastest as the mass load increases for high order resonances. On the other hand, the sensitivity increases the slowest as the mass load increases when *a*/*l* closes to zero.

## 5. Conclusions

The analytical expression for a resonant-mode based bi-layered cantilever with distributed load mass is derived and the mode of vibration, nodal point, resonant frequency, as well as sensitivity can be determined with given mass distribution conditions. Based on the theoretical results, several conclusions are made as follows:

1. Asymmetric mass load distribution causes the nodal points as well as the sensitive regions to shift but the shift value is not related to the degree of symmetry of mass load distribution.
2. There are *n* − 1 local maximal and *n* − 1 local minimal values for the sensitivity changing as mass load length when the cantilever vibrates in the *n*th-order resonance and the maximal sensitivity is found at the first local maximal value.
3. The behavior of mass load length as a function of the maximal sensitivity follows the rule of an exponent decaying function.
4. Sensitivity linearly increases as the load mass increases for the same mass load distribution and behavior of the slopes as a function of mass load length is very similar to that of the sensitivity in the same order resonance but the peak and valley positions are different.

## Figures and Tables

**Table 1:** The cantilever beam material properties and mass load conditions in this study.

Properties	Beam Materials	Symbol	Unit	Value
Young’s modulus	Copper	*E_p_*	GPa	110 [19]
	Metglas 2826	*E_a_*		105 [20]
Density	Copper	*ρ_p_*	kg/m^3^	8.9 × 10^3^ [19]
	Metglas 2826	*ρ_a_*		7.9 × 10^3^ [20]
Poisson’s ratio	Copper	*v_p_*	/	0.36 [19]
	Metglas 2826	*v_a_*	/	0.33 [21]
Length	Copper	*l*	mm	1
	Metglas 2826			
Width	Copper	*w*	mm	0.2
	Metglas 2826			
Thickness	Copper	*t_p_*	μm	15
	Metglas 2826	*t_a_*		
Length ratio of mass load to the cantilever beam	Copper	*a/l*	/	0, 0.1, 0.2, …, 1.0
*ρ_m_A_m_*/*ρ*_0_*A*_0_	/	*r*	/	0.5, 0.6, 0.7, 0.8, 0.9, 1

## References

[1] Johnson B.N., Mutharasan R. (2012). Biosensing using dynamic-mode cantilever sensors: A review. Biosens. Bioelectron..

[2] Zhang K.W., Zhang L., Fu L.L., Li S.Q., Chen H.Q., Cheng Z.Y. (2013). Magnetostrictive resonators as sensors and actuators. Sens. Actuators A Phys..

[3] Sharma H., Mutharasan R. (2013). Rapid and sensitive immuno detection of Listeria monocytogenes in milk using a novel piezoelectric cantilever sensor. Biosens. Bioelectron..

[4] Rosario R., Mutharasan R. (2014). Piezoelectric excited millimeter sized cantilever sensors formeasuring gas density changes. Sens. Actuators B Chem..

[5] Johnson B.N., Mutharasan R. (2013). A cantilever biosensor-based assay for toxin-producing cyanobacteria Microcystis aeruginosa using 16S rRNA. Environ. Sci. Technol..

[6] Zhang K.W., Fu L.L., Zhang L., Cheng Z.Y., Huang T.S. (2014). Magnetostrictive particle based biosensors for in situ and real-time detection of pathogens in water. Biotechnol. Bioeng..

[7] Fu L.L., Zhang K.W., Li S.Q., Wang Y.H., Huang T.S., Zhang A.X., Cheng Z.Y. (2010). In situ real-time detection of *E. coli* in water using antibody-coatedmagnetostrictive microcantilever. Sens. Actuators B Chem..

[8] Fu L.L., Li S.Q., Zhang K.W., Chen I.H., Petrenko V.A., Cheng Z.Y. (2007). Magnetostrictive microcantilever as an advanced transducer for biosensor. Sensors.

[9] Merhaut J. (1981). Theory of Electroacoustics.

[10] Lacheisserie E. (1993). Magnetostriction: Theory and Applications of Magnetoelasticity.

[11] Lochon F., Dufour I., Rebière D. (2005). An alternative solution to improve sensitivity of resonant microcantilever chemical sensors: comparison between using high-order modes and reducing dimensions. Sens. Actuators B Chem..

[12] Jin D.Z., Li X.X., Liu J., Zuo G.M., Wang Y.L., Liu M., Yu H.T. (2011). High-mode resonant piezoresistive cantilever sensors for tens-femtogram resoluble mass sensing in air. J. Micromech. Microeng..

[13] Johnson B.N., Mutharasan R. (2012). Sample preparation-free real-time detection of microRNA in human serum using piezoelectric biosensors at attomole level. Anal. Chem..

[14] Neves M.A.D., Blaszykowski C., Bokhari S., Thompson M. (2015). Ultra-high frequency piezoelectric aptasensor for the label-free detection of cocaine. Biosens. Bioelectron..

[15] Yi J.W., Shih W.Y., Shih W.H. (2002). Effect of length, width, and mode on the mass detection sensitivity of piezoelectric unimorph cantilevers. J. Appl. Phys..

[16] Dohn S., Svendsen W., Boisen A., Hansen O. (2007). Mass and position determination of attached particles on cantilever based mass sensors. Rev. Sci. Instrum..

[17] Maraldo D., Mutharasan R. (2008). Mass-change sensitivity of piezoelectric-excited millimeter-sized cantilever (PEMC) sensors: Model and experiments. Sens. Actuators B Chem..

[18] Zhang K.W., Chai Y.S., Fu J.H. (2015). Study of node and mass sensitivity of resonant mode based cantilevers with concentrated mass loading. AIP Adv..

[19] Read D.T. (1998). Young’s modulus of thin films by speckle interferometry. Meas. Sci. Technol..

[20] The Properties of Metglas 2826. http://www.metglas.com.

[21] Liang C., Morshed S., Prorok B.C. (2007). Correction for longitudinal mode vibration in thin slender beams. Appl. Phys. Lett..

[22] Johnson B.N., Mutharasan R. (2011). A novel experimental technique for determining node location in resonant mode cantilevers. J. Micromech. Microeng..

[23] Zhang K.W., Zhang L., Chai Y.S. (2015). Mass Load Distribution Dependence of Mass Sensitivity of Magnetoelastic Sensors under Different Resonance Modes. Sensors.

